# Comparative transcriptome analysis reveals host-associated differentiation in *Chilo suppressalis* (Lepidoptera: Crambidae)

**DOI:** 10.1038/s41598-017-14137-x

**Published:** 2017-10-23

**Authors:** Haiying Zhong, Fengbo Li, Jianming Chen, Juefeng Zhang, Fang Li

**Affiliations:** 1 State Key Laboratory of Breeding Base for Zhejiang Sustainable Pest and Disease Control, Zhejiang Academy of Agricultural Sciences, Hangzhou, 310021 China; 2 Institute of Plant Protection and Microbiology, Zhejiang Academy of Agricultural Sciences, Hangzhou, 310021 China; 3 Sericultural Research Institute, Zhejiang Academy of Agricultural Sciences, Hangzhou, 310021 China

## Abstract

The striped stem borer, *Chilo suppressalis* Walker (Lepidoptera: Crambidae), is one of the most serious rice pests. Besides attacking rice, it also feeds on an economically important vegetable crop, water-oat *Zizania latifolia*. The species feeding on water-oat has higher growth and survival rate than those on rice, suggesting their success in adaptation to the new host plant. However, little is known about the molecular mechanisms of host plant adaptation. Here we investigated the midgut transcriptome responses of *C. suppressalis* larvae reared on rice and water-oat. A total of 1,633 differentially expressed genes were identified, with a greater number up-regulated on the more delicious new host. The up-regulation of most digestive and detoxification-related genes may be the result of adaptation to the changes in nutritional requirements and toxic chemicals during host shift. In contrast, down-regulation of ribosomal genes may be related to their better development performance when feeding on the new host. In conclusion, our results suggest that transcriptional regulation of genes related to digestion, detoxification and ribosome may play an important role in adaptation of *C. suppressalis* to a new host plant.

## Introduction

The striped stem borer, *Chilo suppressalis* Walker (Lepidoptera: Crambidae), is one of the most serious rice pests in Asia, southern Europe, and northern Africa^1–4^. *C. suppressalis* larvae feed within rice stems, and cause severe yield losses and economic damage annually, particularly in China because of the prevalence of rice cultivation^4–8^. As the center of rice domestication^9^, China has been cultivating rice since 7,000 years ago^10^. In many geographic regions of the country, rice was usually planted in a mosaic fashion or under a crop rotation system with 'Jiaobai', which is also called water-oat (*Zizania latifolia*). Water-oat was domesticated as a vegetable crop from wild *Z. latifolia* approximately 2,000 years ago^5,11^. In recent years, water-oat is widely cultivated in China because of its nutritional and economic importance^11^. Water-oat shares a large number of cultivation areas with rice, thus offering *C. suppressalis* an opportunity for host shift from rice to water-oat. Although *C. suppressalis* also attack other plants, only those feeding on either rice or water-oat can complete their life cycles^12–14^, suggesting their adaptation to the new host plant. However, the mechanisms of host plant adaptation in *C. suppressalis* are still incompletely understood.

*C. suppressalis* feeding on water-oat have higher larval survival rates, shorter larval developmental periods, and higher pupal weight than those on rice^12,15–19^, implying that water-oat is a more suitable host plant for growth and survival of *C. suppressalis* compared with rice. Rice and water-oat are closely related in systematics, but they may possess a different biochemical composition and different secondary metabolic products^11^. Because of adaptation to these two host plants, *C. suppressalis* also showed other variation in ecology and biology, such as the seasonal peak of emergence^12,15,20^, courtship and mating behaviors^19,21–24^, and overwintering biology^18,25–27^. Despite these ecological and biological variations revealed after host shift, the molecular mechanisms underlying host plant adaptation remain unclear.

Previous studies have demonstrated that identifying transcriptional changes associated with host shifts is an important step in understanding the molecular mechanisms of insect adaptation to host plants^28–33^. Transcriptional regulations associated with insects feeding on alternative host plants were commonly detected in differentially expressed genes related to digestion, detoxification, and ribosome^33–37^. However, few studies have examined such mechanisms in *C. suppressalis*. In this study, we investigated transcriptional variation in the *C. suppressalis* larval midgut, the most important organ involved in nutrient acquisition and detoxification in adaptation of insects to host plants^38^. A comparative transcriptome analysis between *C. suppressalis* feeding on rice and that on water-oat was performed to identify differentially expressed genes related to host plant adaptation. This study presents the first exploration of transcriptional changes underlying *C. suppressalis* adaptation to a new host plant.

## Methods

### Specimen collection and rearing

*C. suppressalis* are able to transfer from a rice field to a water-oat field, and vice versa^19,24^, so it is difficult to distinguish the water-oat population (JCS) from the rice population (RCS) of *C. suppressalis* in the adjacent fields^19^. To obtain representative JCS and RCS samples, *C. suppressalis* larvae were collected from the water-oat field in Lishui and the rice field in Yuyao, Zhejiang, China in 2015, where rice and water-oat are exclusively planted. The two fields are approximately 300 kilometers apart. The *C. suppressalis* larvae from the water-oat (also called ‘Jiaobai’) field were reared with fresh water-oat stems, while those from the rice field were reared on rice seedlings. All larvae and plants were kept in an insectarium at 28 ± 1 °C, with a photoperiod of 16 h: 8 h (light/dark), and a relative humidity > 80%. Both the JCS and RCS populations were maintained in the laboratory for three generations before dissection. Midguts of 150 larvae each population were dissected on the second day of the 4th instar and food debris removed. Each 50 midguts were used as one sample. Three replicate samples were taken for each population, immediately frozen in liquid nitrogen and stored at −80 °C for RNA isolation.

### RNA isolation, library construction and sequencing

Total RNA was isolated from the midgut tissues using TRIzol Reagent (Invitrogen, USA) following the manufacturer’s instructions. DNA contaminations were removed from the RNA samples by RNase-free DNase I (New England Biolabs, MA, USA). The purity and quantity of total RNA was assessed by a NanoPhotometer^®^ spectrophotometer (IMPLEN, CA, USA) and a Qubit^®^ 2.0 Flurometer (Life Technologies, CA, USA), respectively. RNA integrity was checked using the RNA Nano 6000 Assay Kit of the Agilent Bioanalyzer 2100 system (Agilent Technologies, CA, USA). The total high-quality RNA isolated from three independent biological replicate samples for each *C. suppressalis* population was used individually to construct cDNA libraries. The transcriptome libraries were generated using NEBNext^®^ Ultra™ RNA Library Prep Kit for Illumina^®^ (NEB, USA) according to manufacturer’s recommendations and index codes were added to attribute sequences to each sample. Briefly, the poly-A tailed mRNA were purified with poly-T oligo-attached magnetic beads and fragmented to obtain RNA fragments. Double-stranded cDNA was synthesized from the fragmented RNA. In order to select the cDNA fragments of preferentially 150 bp in length, the library fragments were purified with AMPure XP system (Beckman Coulter, Beverly, USA). Clustering of the index-coded samples was performed on a cBot Cluster Generation System using TruSeq PE Cluster Kit v3-cBot-HS (Illumina) following the manufacturer’s instructions. After clustering, each library was sequenced using Illumina Hiseq. 2000 platform and 150-bp paired-end reads were generated.

### Transcriptome assembly and annotation

Raw sequences were deposited in the NCBI Short Read Archive (SRA) database (http://www.ncbi.nlm.nih.gov/Traces/sra/). Raw reads in FASTQ format were first filtered by removing reads containing adapter sequences and low-quality reads that contain > 10% poly-N or > 50% of bases whose Qphred quality scores ≤ 20. At the same time, Q20, Q30, GC-content and sequence duplication level of the clean data were calculated. Only the clean data with high quality were used for downstream analyses. Transcriptome assembly was accomplished using Trinity software^39^ with min_kmer_cov set to 2 by default and all other parameters set default. Gene function was annotated by homology searches against the NCBI non-redundant protein (Nr) database, NCBI nucleotide (Nt) database, Swiss-Prot protein database, euKaryotic Orthologous Groups (KOG), Kyoto Encyclopedia of Genes and Genomes (KEGG), Gene Ontology (GO), and Protein family (Pfam) database.

### Differential gene expression analysis

Gene expression levels for each sample were estimated by RSEM program^40^. Gene expression was measured in the expected number of Fragments Per Kilobase of transcript sequence per Million base pairs sequenced (FPKM). Differential expression analysis of the two *C. suppressalis* populations was performed using DESeq.^41^, which provides statistical routines for determining differential expression in digital gene expression data using a model based on the negative binomial distribution. The resulting *p-*values were adjusted using the Benjamini and Hochberg’s approach for controlling the false discovery rate. Genes with an adjusted *p*-value < 0.05 detected by DESeq were assigned as differentially expressed. Then GO enrichment analysis of the differentially expressed genes (DEGs) was implemented by GOseq.^42^, while KOBAS^43^ software was used to test the statistical enrichment of DEGs in KEGG pathways.

### qRT-PCR analysis

To confirm the results of transcriptome comparisons, real-time quantitative PCR (qRT-PCR) was performed on randomly selected DEGs using a LightCycler 480 II system (Roche Diagnostics GmbH, Mannheim, Germany) with LightCycler 480 SYBR Green I Master Mix (Roche Diagnostics GmbH, Mannheim, Germany). The amplifications were carried out in a total volume of 20.0 μL and included 10.0 μL of 2X SYBR Green MasterMix reagent, 2.0 μL of cDNA and 0.1 μL of each primer (100.0 μM). The thermal cycling profile was an initial denaturation at 95 °C for 10 min followed by 45 cycles of denaturation at 95 °C for 15 s and annealing/extension at 60 °C for 30 s. The primers for qRT-PCR are listed in Supplementary Table S7. All reactions were conducted in triplicate and included negative controls with no template. The *actinA1* gene was used for normalization in subsequent analysis. The relative expression was analysed using the *R* = 2^−ΔΔ*C*T^ method^44^.

## Results

### Transcriptome sequencing

Insect midgut plays key roles in nutrient digestion and detoxification. The midguts of the 4th-instar larvae reared on rice and water-oat were isolated and used for total RNA isolation. Three biologically independent replicates for each treatment were used for constructing cDNA libraries. Each library was sequenced on the Illumina HiSeq. 2000 platform using paired-end150 bp reads. We sequenced six transcriptome libraries, which were from *C. suppressalis* population reared on rice (RCS1, RCS2, and RCS3) and those reared on water-oat (JCS1, JCS2, and JCS3). High-throughput sequencing resulted in a mean of 71,706,401 reads for each library (Table 1). After data filtering, we obtained 64–82 million clean reads comprised of 9–12 billion nucleotides (9–12 GB) and the sequencing quality was high with Q30 ratio larger than 93% for all the samples. All the data have been deposited at NCBI SRA database (SRP097007).

**Table 1:** Summary of the transcriptome sequencing data from the JCS and RCS samples.

Sample	Raw Reads	Clean reads	Clean bases	Error(%)	Q20(%)	Q30(%)	GC(%)
JCS1	67702014	65610300	9.84 G	0.01	97.28	93.40	45.41
JCS2	68008986	65834344	9.88 G	0.01	97.17	93.18	45.36
JCS3	66544762	64466234	9.67 G	0.01	97.28	93.40	45.58
RCS1	85366504	82708936	12.41 G	0.01	97.27	93.40	44.41
RCS2	73258546	70946782	10.64 G	0.01	97.30	93.46	44.86
RCS3	69357594	67178592	10.08 G	0.01	97.35	93.54	44.82

### *De novo* assembly of the transcriptome

We pooled all the high-quality clean reads from six libraries and *de novo* assembled them using Trinity software with the default parameters. A total of 416,745,188 clean reads were randomly assembled to produce 177, 596 contigs with a N50 of 1702 bp and a mean length of 848 bp. The contigs were further assembled into 126,323 unigenes with a N50 of 1103 bp, a mean length of 639 bp, a minimum length of 201 bp and a maximum length of 34,432 bp (Table 2). There were 40,795 (22.97%) contigs and 18,125 (14.34%) unigenes more than 1000 bp. The detailed length distribution of contigs and unigenes was shown in Supplementary Fig. S1.

**Table 2:** Assembly statistics for the midgut tissue transcriptome of *Chilo suppressalis*.

	Min Length	Mean Length	Median Length	Max Length	N50	N90	Total
Transcripts	201	848	386	34432	1702	291	177596
Unigenes	201	639	316	34432	1103	247	126323

### Functional annotation

After transcriptome *de novo* assembly for the *C. suppressalis* midgut tissue samples, the unigenes were used for functional annotation. Of the 126,323 unigenes, 43,570 (34.49%) had at least one significant hit against at least one of the seven databases, which include Nr, Nt, Pfam, Swiss-Prot, KOG, KEGG, and GO (Table 3 and Fig. S2). A total of 32,788 unigenes (25.95% of all the unigenes) had significant hits against the Nr database. Among these unigenes, approximately 16,361 showed top matches with unigenes from silkworm *Bombyx mori* (23.4%), followed by monarch butterfly *Danaus plexippus* (13.5%) and diamondback moth *Plutella xylostella* (13.0%) (Fig. S3). This result was consisted with the evolutionary relationship as *C. suppressalis* is a representative of lepidopterans.

**Table 3:** Summary statistics of function annotation.

	Number of Unigenes	Percentage (%)
Annotated in Nr	32788	25.95
Annotated in Nt	17954	14.21
Annotated in KEGG	13374	10.58
Annotated in Swiss-Prot	22266	17.62
Annotated in Pfam	24047	19.03
Annotated in GO	24385	19.3
Annotated in KOG	14959	11.84
Annotated in all Databases	5200	4.11
Annotated in at least one Database	43570	34.49
Total Unigenes	126323	100

**Figure 1:**
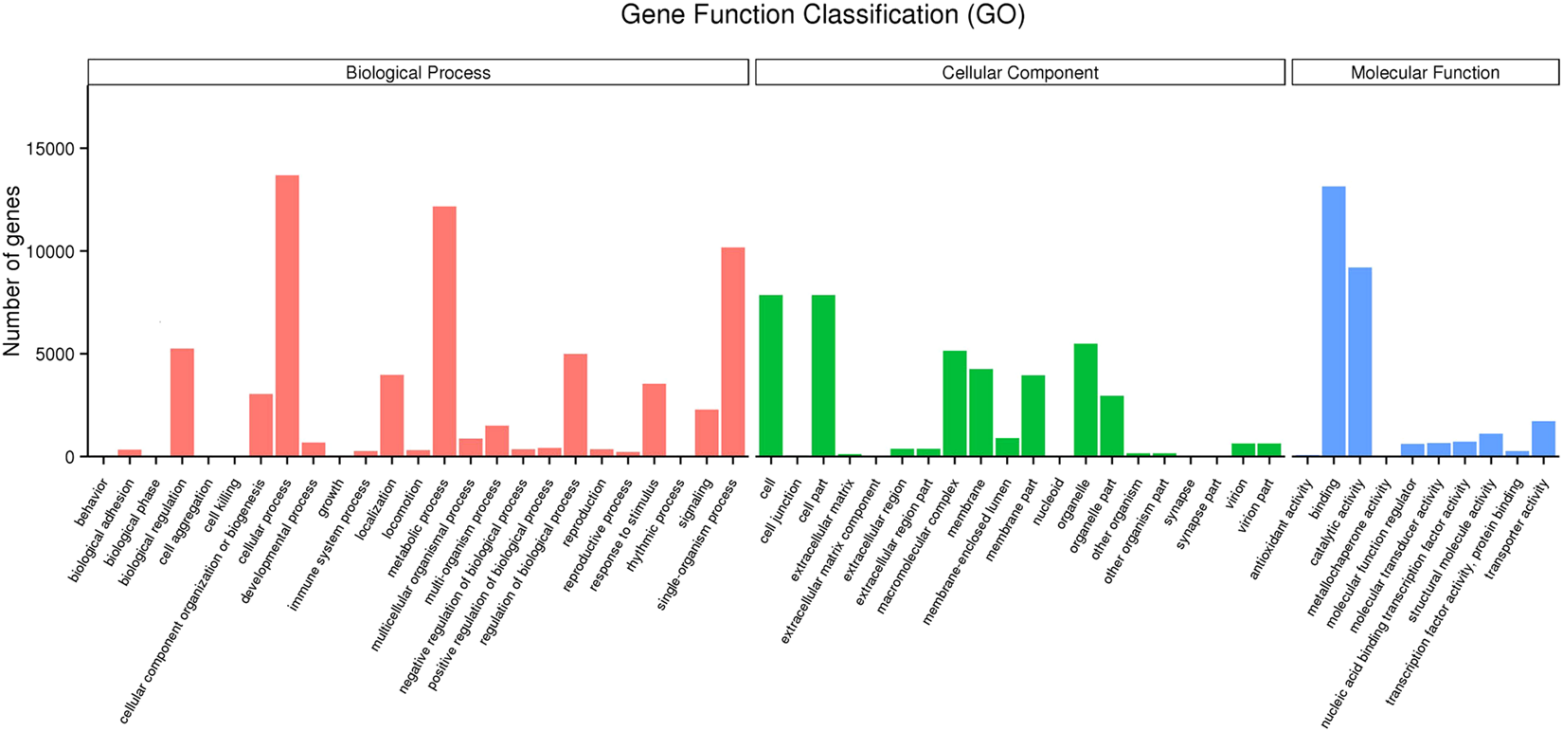
Gene Ontology (GO) classification of transcripts of midgut tissue samples. Go categories, shown in the x-axis, are assigned into three main ontologies: biological process, cellular component, and molecular function. The y-axis indicates the number of unigenes in each category.

GO annotation was performed based on the Nr and Pfam annotation results using Blast2GO^45^. A total of 24,385 unigenes (19.30%) were assigned to GO terms (Table 3). As shown in Fig. 1, the unigenes were categorized into 55 GO terms, including 25 (45.45%) biological process terms, 20 (36.36%) cellular component terms and 10 (18.18%) molecular function terms. For the biological process category, cellular process (13,687) was the most predominant term, followed by metabolic process term (12,176) and single-organism process term (10,182). In the cellular component category, cell (7,849) and cell part terms (7,849) were the predominant terms and followed by organelle term (5,497) and macromolecular complex term (5,147). Within the molecular function category, binding (13,148) was the most abundant term, followed by catalytic activity term (9,198) and transporter activity term (1,721).

**Figure 2:**
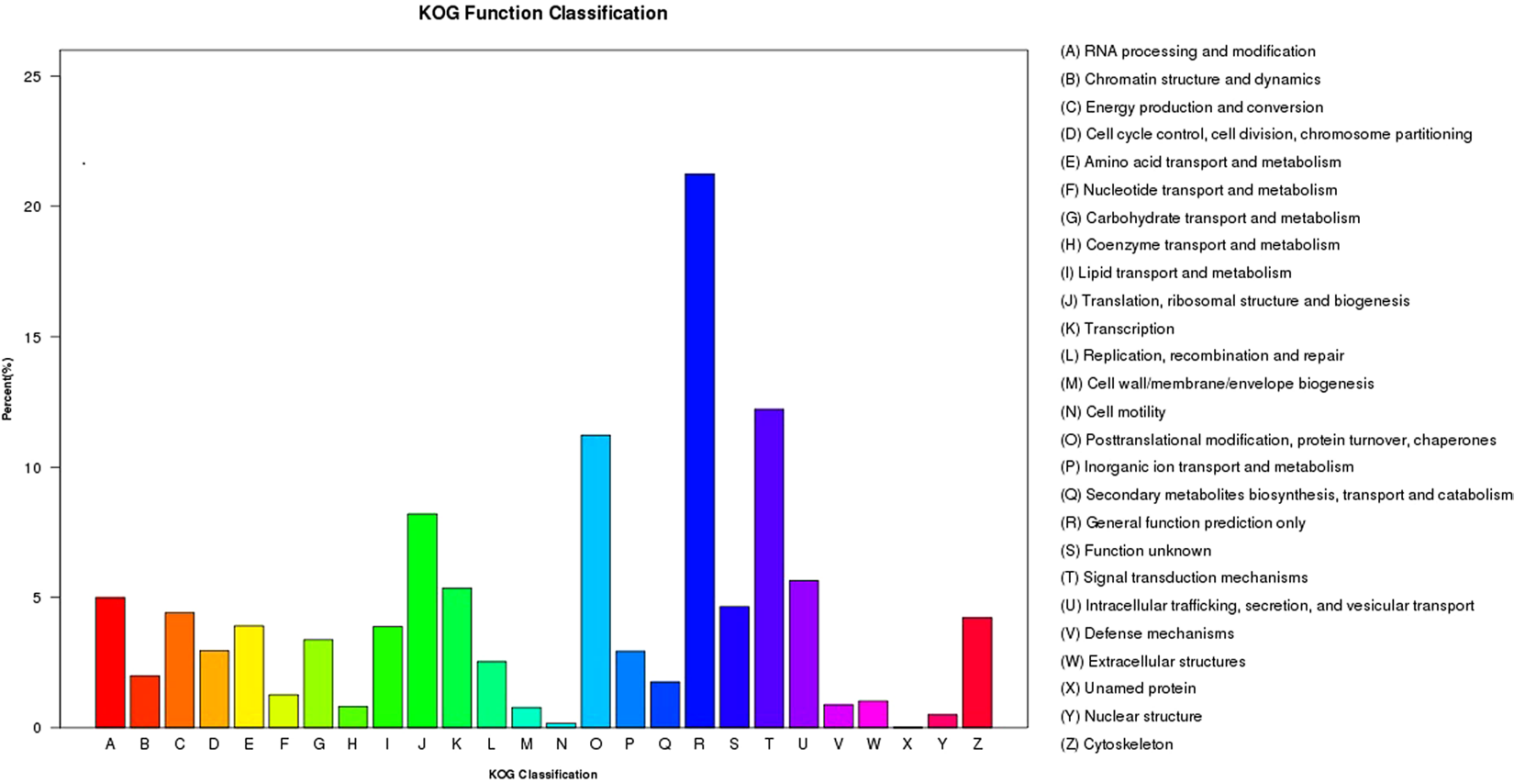
euKaryotic Ortholog Groups (KOG) classification of unigenes.

Further, the unigene sequences of *C. suppressalis* were searched against the KOG database for functional prediction and classification. A total of 14,959 unigenes (11.84% of all the unigenes) were assigned into 26 different KOG categories (Fig. 2). Among the KOG categories, the largest group was the “general function prediction only” (3,177 unigenes, 21.23%), followed by “signal transduction mechanisms” (1,829 unigenes, 12.22%), “posttranslational modification, protein turnover, chaperones” (1,680 unigenes, 11.23%), and “translation, ribosomal structure and biogenesis” (1,228 unigenes, 8.20%) (Fig. 2) (see Supplementary Table S1). Only a few unigenes were assigned to “nuclear structure” (0.50%), “cell motility” (0.16%), and “unamed protein” (less than 0.01%), which represent the smallest groups.

To identify the biological pathway in *C. suppressalis* transcriptome, all unigenes were mapped to the KEGG database. As a result, a total of 13,374 unigenes were classified into 230 KEGG pathways (Table S2). Among these KEGG pathways, the top 50 pathways with the largest group of unigenes are shown in Supplementary Table S3.

### ORF prediction

**Figure 3:**
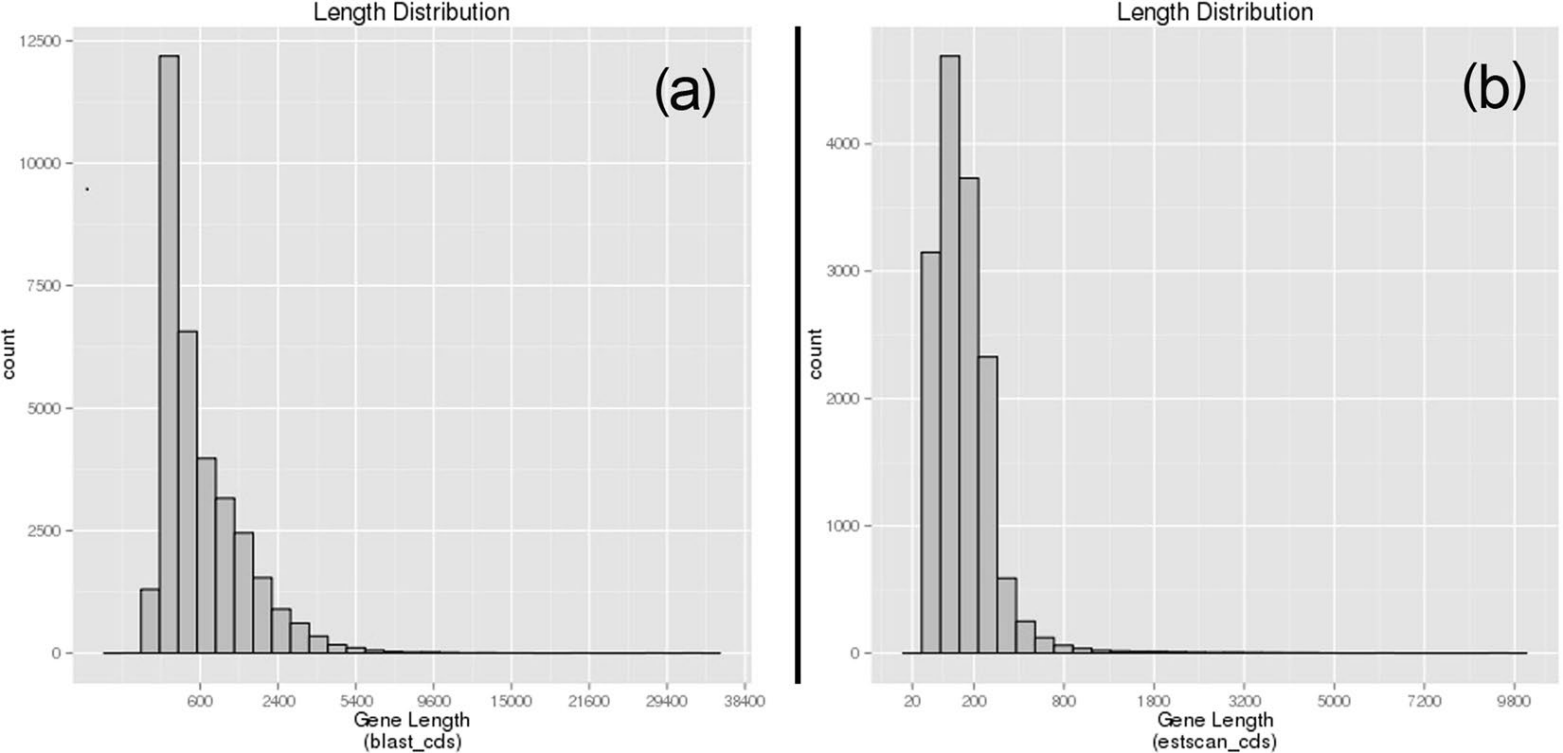
Length distribution of ORF prediction. (**a**) Length distribution of ORF predicted in the Nr and Swiss-Prot databases, (**b**) Length distribution of ORF predicted using the estscan software.

Based on the BLASTx results compared with Nr and Swiss-Prot databases, the Open Reading Frame (ORF) was predicted from the annotated unigenes, with an average ORF length of 784 bp ranging from 27 bp to 32,973 bp (Fig. 3a). For the unigenes from which no ORF was successfully detected within the 2 databases, the estscan software was used to identify potential ORF. A total of 15,057 unigenes were predicted containing ORF, with an average length of 178 bp ranging from 51 bp to 9,252 bp (Fig. 3b).

### Comparative analysis of the midgut transcriptome

**Figure 4:**
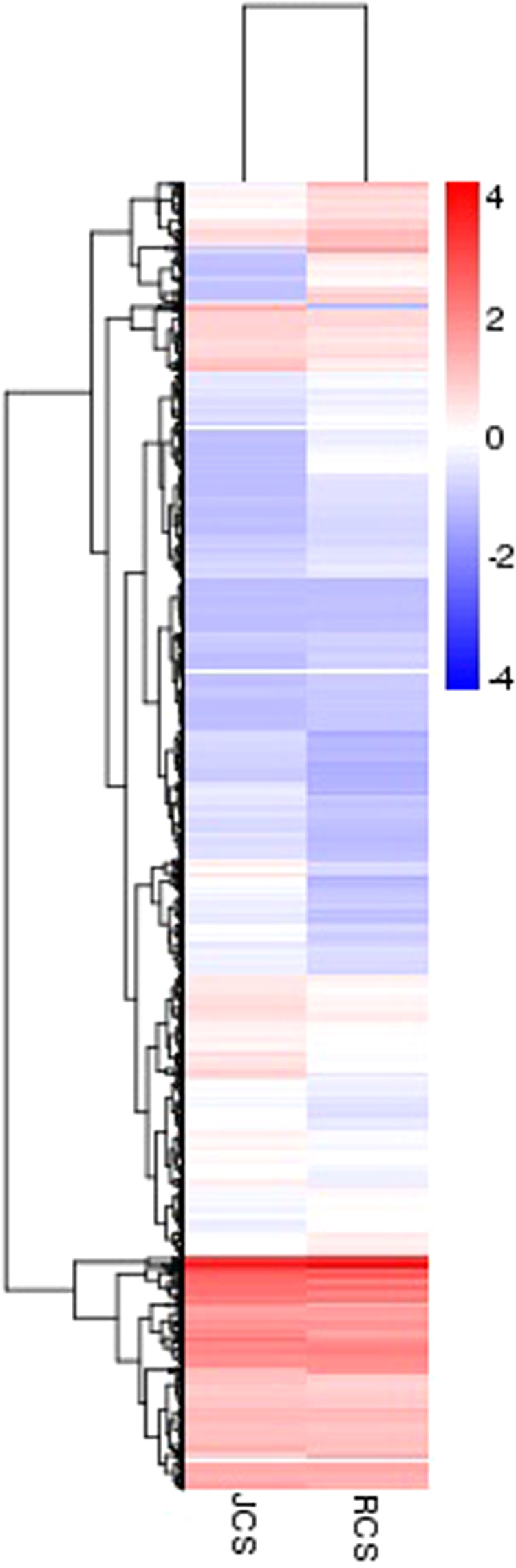
A hierarchical clustering of DEGs based on log_10_ (FPKM+1) values in RCS and JCS. The red bands indicate high gene expression levels, and the blue bands indicate low gene expression levels.

To understand the molecular mechanisms of host plant adaptation in *C. suppressalis*, we performed a comparative analysis of six midgut transcriptomes. Based on the adjusted *p*-value < 0.05, we identified 1,633 differentially expressed genes (DEGs) in JCS compared with RCS, which include 932 up-regulated genes and 701 down-regulated genes (Fig. S4). These DEGs, listed in the Supplementary Table S4, were mainly involved in midgut physiological functions such as digestion, detoxification, transport, and metabolism. The commonly reported detoxification-related DEGs, such as P450s, GSTs, UGTs, and CEs, were able to be detected. All of them were mainly up-regulated. The overall pattern of midgut gene expression between JCS and RCS, presented in the hierarchical clustering heatmap (Fig. 4), further showed that a large number of genes were regulated in adaptation to a new host plant.

**Figure 5:**
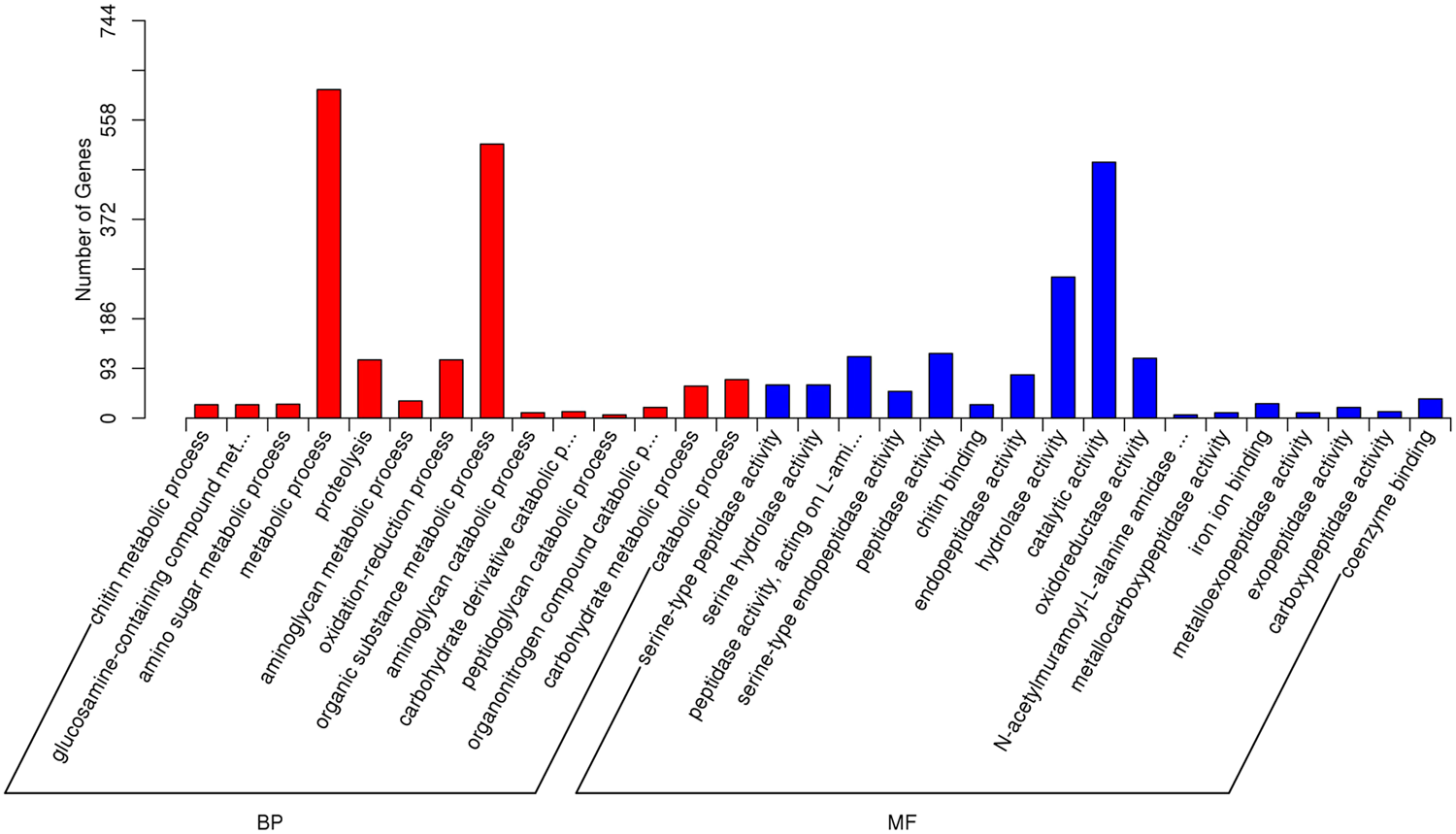
GO enrichment analysis of the differentially expressed unigenes.

To analyze functions of the DEGs in the midgut, GO term enrichment analysis was performed using the software Blast2GO. The result shows the strongest changes in adaptation to a new host were in the metabolic process (615 DEGs) and organic substance metabolic process (513 DEGs) of biological process categories, followed by the catalytic activity (479 DEGs) and hydrolase activity (264 DEGs) of molecular function categories (Fig. 5). Enriched GO terms in the midgut were mainly involved in digestive, detoxifying, and metabolism-related functions (Table S5). Within the GO terms, most DEGs were up-regulated (Fig. S5a,b), suggesting that a large number of genes were mainly up-regulated in adaptation to a novel host plant in *C. suppressalis*. Then we performed GO term enrichment analysis for the up-regulated and down-regulated DEGs, respectively. The results shows that there are more DEGs up-regulated especially in the molecular function category (Fig. S5b). In the cellular component category, however, no DEG up-regulated was detected, while some DEGs related to ribosome were obviously down-regulated (Fig. S5c). The results imply that down-regulation of DEGs related to ribosome may contribute to the host plant adaptation in *C. suppressalis*.

**Figure 6:**
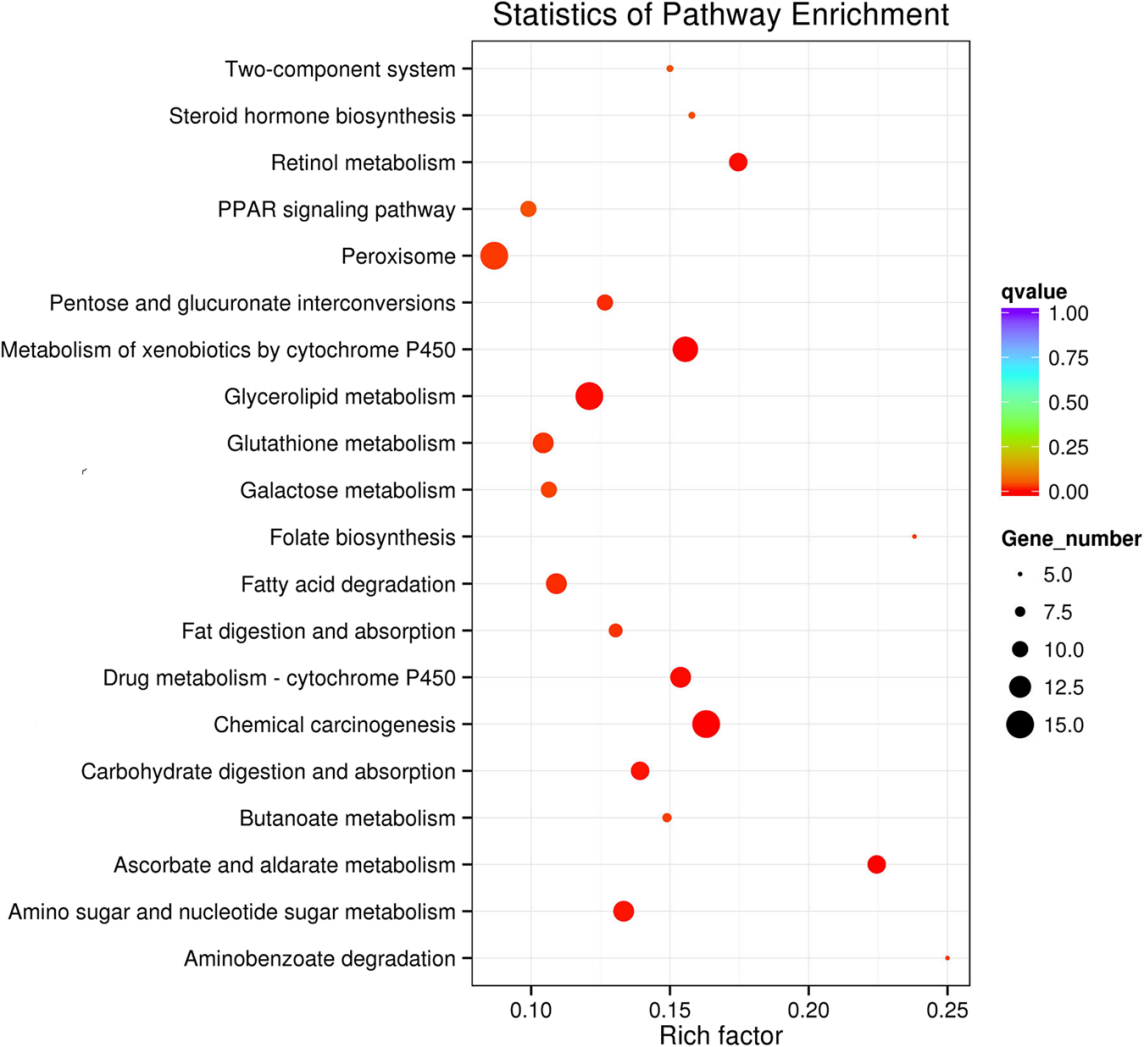
Scatterplot of enriched KEGG pathways for DEGs. The enrichment factor indicates the ratio of the DEG number to the total gene number in a certain pathway. The color and size of dots indicate the range of *q*-value and gene number, respectively.

To obtain more insight into the possible functions of the DEGs, the KEGG pathway analysis were carried out using KOBAS 2.0. The result shows that the DEGs in the JCS midgut were involved in 20 KEGG pathways (Table S6). The pathways related to digestion, detoxification, nutrient metabolism, and growth were over-represented (Fig. 6). For example, the significantly enriched digestion pathways mainly included “Carbohydrate digestion and absorption” and “Fat digestion and absorption”. The significantly enriched detoxification pathways mainly included “Drug metabolism-cytochrome P450”, “Metabolism of xenobiotics by cytochrome P450”, and “Glutathione metabolism” (Table S6). The similar pathways were found over-represented in the up-regulated DEGs (Fig. S6a), but the down-regulated DEGs were significantly enriched to a different pathway related to “Ribosome” (Fig. S6b), suggesting that the “Ribosome” pathway may play a key role in the host plant adaptation in *C. suppressalis*.

**Figure 7:**
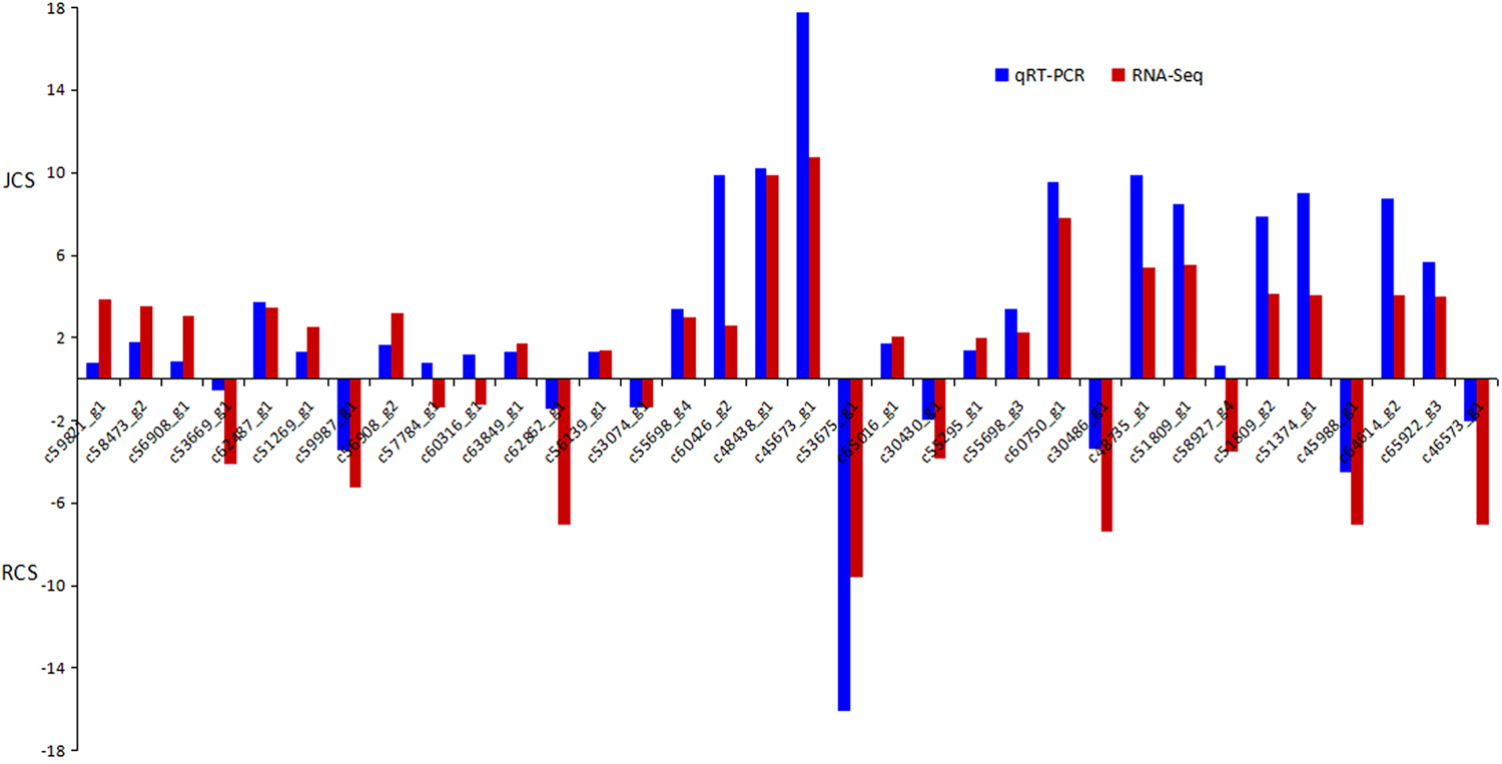
Comparison of gene expression patterns obtained by RNA-Seq and qRT-PCR. Log-fold changes are expressed as the ratio of gene expression after normalization to *actinA1*.

To validate the DEGs identified by comparative transcriptomic analysis, we randomly selected 34 genes for qRT-PCR. Among them, 21 were digestive, detoxifying, and ribosome-related genes. As shown in Fig. 7, the qRT-PCR expression patterns of 31 out of 34 randomly selected DEGs were in agreement with the results of the transcriptome analysis.

## Discussion

Differences in nutritional value between host plants might require herbivorous insects to express different digestive enzymes^33,46,47^. We anticipated that digestion-related genes of *C. suppressalis* might be regulated in adaptation to different plant hosts. In this study, a total of 83 digestion-related genes were differentially expressed in *C. suppressalis* larvae in adaptation to a novel host plant (Table 4; Supplementary Table S4 in detail). Among these DEGs, 79 were up-regulated, whereas only four down-regulated. Ge *et al*. reported previously that the expression of three digestive cysteine protease genes in *C. suppressalis* was significantly affected by the host plant type^48^. Although only one DEG (c60073_g1) belonging to cysteine protease family was identified in our study, it was significantly up-regulated in *C. suppressalis* larvae feeding on water-oat in comparison with those feeding on rice. This result is consistent with the previous study^48^. Another DEG (c53074_g1) in the digestive gene list was found to be the same as the serine protease inhibitor gene *CS003*, which had been identified in *C. suppressalis* midgut^49^. It’s worth noting that both *CS003* and c53074_g1 showed similar down-regulated expression in *C. suppressalis* larvae feeding on water-oat compared with those feeding on rice, although quantitative gene expression assays were performed using qRT-PCR in the earlier study and RNA-Seq in this study. We also identified a large number of serine proteases, which are recognized as key enzymes allowing larvae to adapt to different diets^33,35,50^, and observed their differential expression regulation in response to different diets. These findings further emphasize the role of digestive genes (such as cysteine proteases, serine protease inhibitors, and serine proteases) in host plant adaptation. In addition, another 54 genes that correspond to carboxypeptidase, aminopeptidase, dipeptidase, α-amylase, α-glucosidase and lipase (Table 4) are also worthy to be investigated in the future. These digestion-related genes might play important roles in adaptation of *C. suppressalis* to a novel host plant.

**Table 4:** Summary of candidate differentially expressed genes related to digestion, detoxification and ribosome in *Chilo suppressalis* gut transcriptome.

Classification	Candidate genes	Number of DEGs		
		Total	Up	Down
**Digestion**	Serine protease	27	25	2
	Serine protease inhibitor	1	0	1
	Lipase	12	11	1
	Carboxypeptidase	16	16	0
	Cystein protease	1	1	0
	Aminopeptidase	15	15	0
	Dipeptidase	4	4	0
	α-amylase	5	5	0
	α-glucosidase	2	2	0
**Detoxification**	P450s	16	16	0
	CEs	13	13	0
	GSTs	7	6	1
	UGTs	2	1	1
	ABC transporters	5	1	4
**Ribosome**	Ribosomal protein	42	0	42

Besides nutritional requirements, phytophagous insects need to cope with toxic chemicals from their host plants. Their ability to detoxify these chemicals may determine their host range. The detoxification process is generally divided into three phases involving in a set of detoxification-related enzymes and xenobiotic transporters^30,33–35,37,47^. Phase I enzymes, including cytochrome P450 proteins (P450s) and carboxylesterases (CEs), participate in direct metabolism of xenobiotics, while phase II enzymes, including glutathione S-transferases (GSTs) and UDP-glycosyltransferases (UGTs), transform allelochemicals into water-soluble compounds for excretion by ATP binding cassette (ABC) transporters or sequestration during phase III. RNA-Seq has proved to be an effective approach to identify detoxification genes related to host plant adaptation^30–33,35,47^. In this study, we used RNA-Seq to identify 42 DEGs related to detoxification (Table 4). Among these DEGs, all the phase I enzymes (P450s and CEs) and most of phase II enzymes (GSTs and UGTs) were up-regulated in *C. suppressalis* larvae feeding on water-oat compared with those feeding on rice, suggesting that these genes might have important roles in detoxification during host plant adaptation. In contrast, only one member of ABC transporters, subfamily A, was up-regulated, whereas the other ABC transporters including subfamilies B and C were mainly down-regulated, highlighting the potential role of ABC transporters in detoxification. Similar results were observed in other insects, where members of subfamilies B and C, involved in detoxification and multidrug resistance, were differentially regulated in adaptation to a host plant^29,37,51^. These results, together with higher larval survival rates^19^, suggest that *C. suppressalis* may succeed in feeding on water-oat by detoxifying toxic chemicals in which the upregulation of the phase I and II enzymes and the downregulation of ABC transporters could play an important role.

In the present study, the most prominent change in gene expression of *C. suppressalis* in adaptation to a novel host plant is in the ribosome. The results of GO and KEGG enrichment analysis indicated that DEGs related to ribosome were specifically over-represented and all down-regulated in *C. suppressalis* larvae feeding on water-oat, suggesting the role of genes coding ribosomal proteins in host plant adaptation. Ribosomes are highly conserved molecular machines whose gene expression has traditionally been considered to be stable and function mainly in protein translation. However, recent studies have reported that some ribosomal genes were differentially regulated in response to different host plants in many insect species, such as tobacco budworm *Heliothis virescens* ^52^, cotton bollworm *Helicoverpa armigera* ^28^, whitefly *Bemisia tabaci* ^53^, butterfly *Polygonia calbum* ^35^, and ladybird *Cryptolaemus montrouzieri* ^54^. Of particular interest is the upregulation of these ribosomal genes when insects fed on unsuitable diets^28,35,52–54^. *C. suppressalis* feeding on water-oat had higher survival rate, shorter larval developmental periods, larger bodied larvae, pupae and adults, as compared with those feeding on rice^12,15,17–19^, implying that water-oat may be a more suitable diet for *C. suppressalis*. Thus this well explains why ribosomal genes were down-regulated in *C. suppressalis* larvae feeding on water-oat. These differential regulations in insects have been considered to counteract ribosome-inactivating proteins (RIPs) in plants, which involved in insecticidal activity^35,55^. No RIPs has been reported in water oat, but a genome-wide survey has identified 31 RIPs in rice^56^. In the future, a similar genome-wide identification of RIPs in water-oat will facilitate our better understanding of the molecular mechanisms in insect adaptation to a host plant.

Besides host plant, environmental factors or natural genetic variation might affect gene expression patterns observed owing to our sampling strategy. A large number of studies, however, have demonstrated that *C. suppressalis* diverged into rice population (RCS) and water-oat population (JCS) because of adaptation to different host plants^12,18,19,24–27^. Our investigations also suggest that the biological differences between RCS and JCS were affected mainly by host plant^16^,unpublisheddata. Especially, previous studies have showed that there is high genetic similarity among *C. suppressalis* populations in different distribution areas^57–60^. Additionally, similar sampling strategies have been documented in previous reports^19,61–64^. We thus think that the different gene expression patterns observed in our study may be caused mainly by host plant.

In conclusion, RNA-seq analysis provided an initial step toward improving our understanding of the molecular mechanisms underlying *C. suppressalis* adaptation to a new host plant at the transcriptome level. The present study revealed a large number of DEGs involving in host plant adaptation of *C. suppressalis*. Our next study will testify if the differential gene expression is truly associated with characters of host plants (such as nutrient), and verify the exact functions of these genes individually.

## Electronic supplementary material

Supplementary Information (Figures S1-S6, Tables S1-S7)

Table S1

Table S2

Table S3

Table S4

Table S5

Table S6

Table S7
