# Supplementary material for: Comparative transcriptome analysis reveals host-associated differentiation in Chilo suppressalis (Lepidoptera: Crambidae)
Source: Sci Rep. 2017 Oct 23;7:13778. doi: 10.1038/s41598-017-14137-x (PMC5653757; doi:10.1038/s41598-017-14137-x)
Supplement: Supplementary file 1 — Supplementary Information (Figures S1-S6, Tables S1-S7) [file 41598_2017_14137_MOESM1_ESM.doc]

**Supplementary Information** **(Figures S1-S6, Tables S1-S7)**

**Comparative transcriptome analysis reveals host-associated differentiation in *Chilo suppressalis* (Lepidoptera: Crambidae)**

**Haiying Zhong**^1,2^, **Fengbo Li**^1,3,*^, **Jianming Chen**^1,2,*^, **Juefeng Zhang**^1,2^, **Fang Li**^1,2^

^1^ State Key Laboratory of Breeding Base for Zhejiang Sustainable Pest and Disease Control, Zhejiang Academy of Agricultural Sciences, Hangzhou 310021, China.

^2^ Institute of Plant Protection and Microbiology, Zhejiang Academy of Agricultural Sciences, Hangzhou 310021, China.

^3^ Sericultural Research Institute, Zhejiang Academy of Agricultural Sciences, Hangzhou 310021, China.

Emails: [zhy8085@nwsuaf.edu.cn](mailto:zhy8085@163.com) (HaiyingZhong), [fengboli@gmail.com (fengboli](mailto:fengboli@hotmail.com(fengboli) Li), jianmchen63@163.com (Jianming Chen)

^*^Correspondings: Chen Jianming, Li Fengbo

Phone numbers: +86-571-86400486;+86-571-86404173

Email addresses:jianmchen63@163.com (Jianming Chen); [fengboli@gmail.com](mailto:fengboli@hotmail.com)(fengbo Li)

**Supplementary Information:**

**Figure S1. Distribution of the length of all the assembled transcripts and unigenes in *C. suppressalis.***

**Figure S2. Function annotation of unigenes in the five main public databases (Nr, Nt, KOG, Pfam, and GO).**

**Figure S3. Characteristics of the homology search results for the unigenes against the Nr database.** (**a**) Species distribution is shown as a percentage of the total homologous sequences with an E-value cutoff 1.0E-5. (**b**) E-value distribution of the BLAST hits for each unique sequence with a cutoff E-value < 10^-5^. (**c**) Similarity distribution of the top BLAST hits for each sequence.

**Figure S4. Transcriptional differences between *C. suppressalis* reared on rice (RCS) and water-oat (JCS).** The fold changes on x-axis represent the ratio of transcript abundance of *C. suppressalis* on water-oat and rice, respectively. Differentially expressed transcripts are highlighted in red [*q* < 0.05, Log_2_ (fold change) >1] and blue [*q* < 0.05, Log_2_ (fold change) < -1], respectively on the volcano plot. Marked in red are the transcripts with a significant differentiae in the high expression level; marked in green are the transcripts with a significant differentiae in the low expression level; and marked in blue are the transcripts without significant differentiae.

**Figure S5. GO enrichment analysis of DEGs (JCS vs RCS).** (**a**) The up- and down-regulated DEGs. (**b**) The up-regulated DEGs. (**c**) The down-regulated DEGs. The x-axis shows the GO terms, and the y-axis indicates the number of genes.

**Figure S6. Scatterplot of enriched KEGG analysis of the up- and down-regulated DEGs.** (**a**) The up-regulated DEGs. (**b**) The down-regulated DEGs. The enrichment factor indicates the ratio of the DEG number to the total gene number in a certain pathway. The color and size of dots indicate the range of *q*-value and gene number, respectively.

**Table S1.** KOG_classification.

**Table S2.** KEGG_classification.

**Table S3.** The top 50 KEGG pathways with the largest group of unigenes.

**Table S4.** Differentially expressed genes (DEGs).

**Table S5.** GO enrichment results of DEGs.

**Table S6.** KEGG pathway enrichment results of DEGs.

**Table S7.** Primers used in validation experiment of gene expression by qRT-PCR.


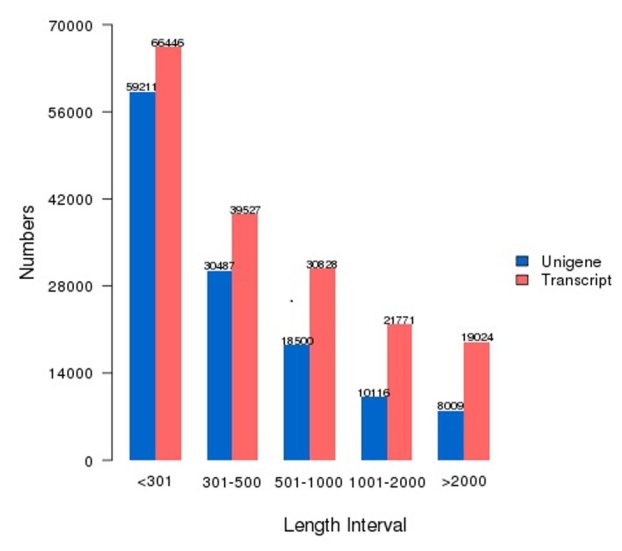


**Figure S1.** Distribution of the length of all the assembled transcripts and unigenes in *C. suppressalis.*


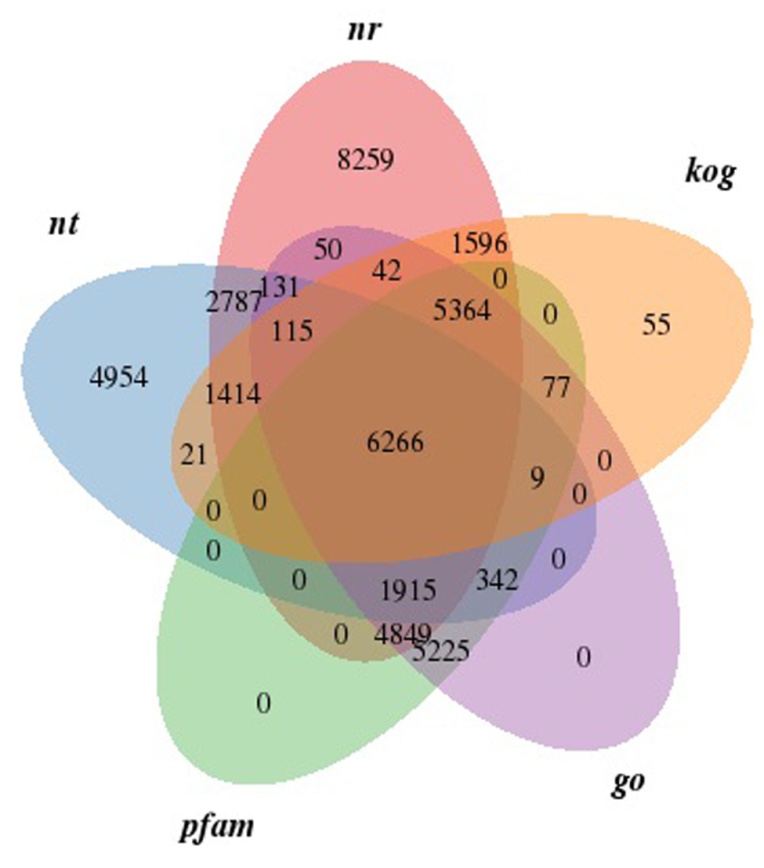


**Figure S2.** Function annotation of unigenes in the five main public databases (Nr, Nt, KOG, Pfam, and GO).


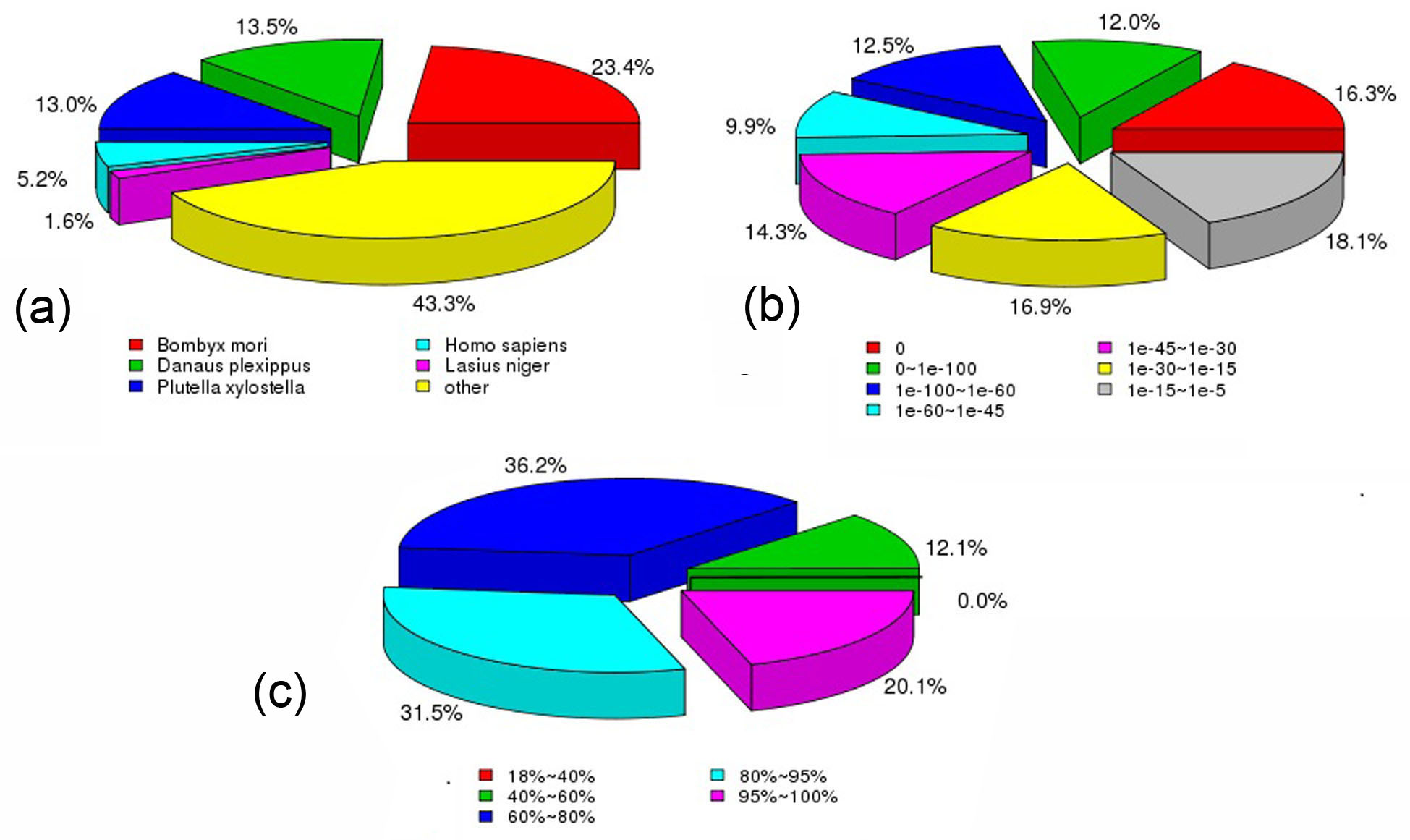


**Figure S3.** Characteristics of the homology search results for the unigenes against the Nr database. (**a**) Species distribution is shown as a percentage of the total homologous sequences with an E-value cutoff 1.0E-5. (**b**) E-value distribution of the BLAST hits for each unique sequence with a cutoff E-value < 10^-5^. (**c**) Similarity distribution of the top BLAST hits for each sequence.


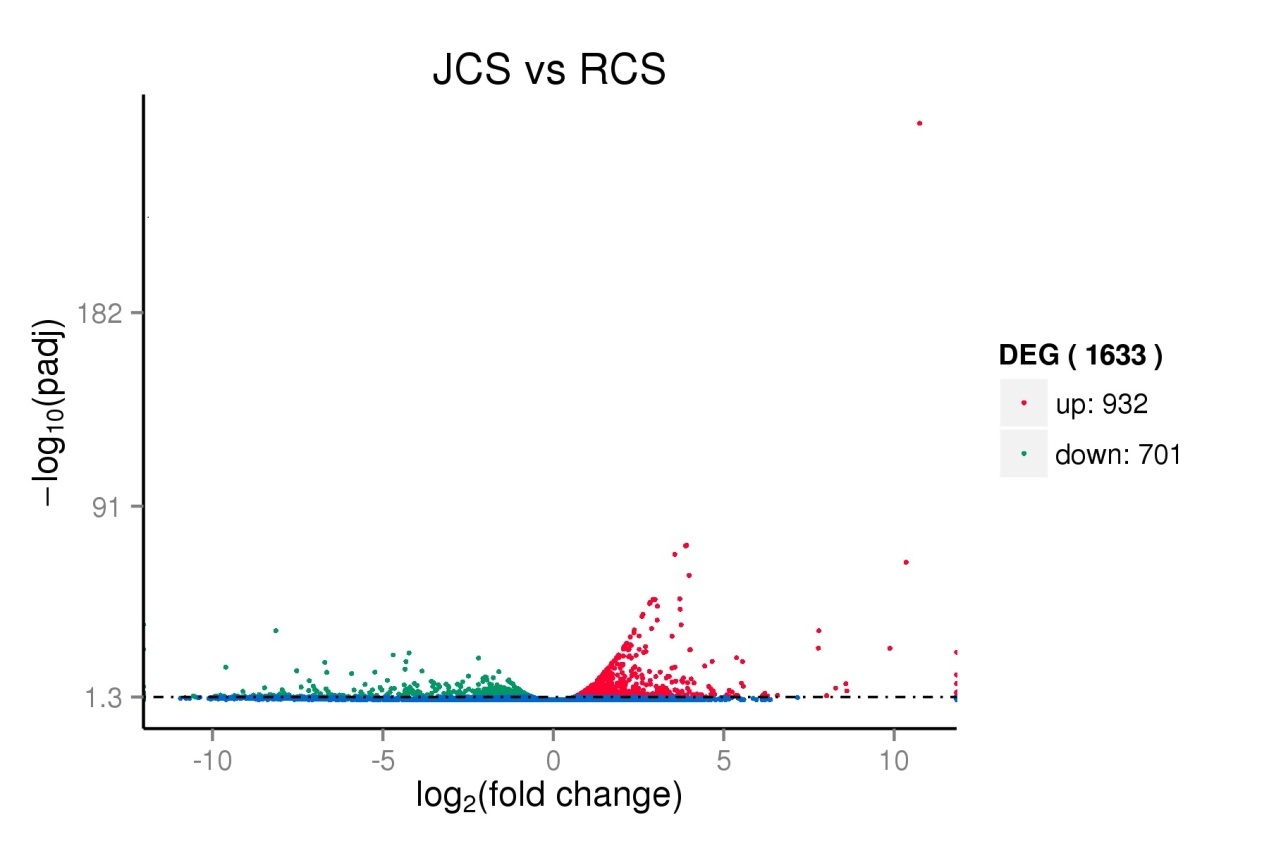


**Figure S4.** Transcriptional differences between *C. suppressalis* reared on rice (RCS) and water-oat (JCS). The fold changes on x-axis represent the ratio of transcript abundance of *C. suppressalis* on water-oat and rice, respectively. Differentially expressed transcripts are highlighted in red [*q* < 0.05, Log_2_ (fold change) >1] and blue [*q* < 0.05, Log_2_ (fold change) < -1], respectively on the volcano plot. Marked in red are the transcripts with a significant differentiae in the high expression level; marked in green are the transcripts with a significant differentiae in the low expression level; and marked in blue are the transcripts without significant differentiae.


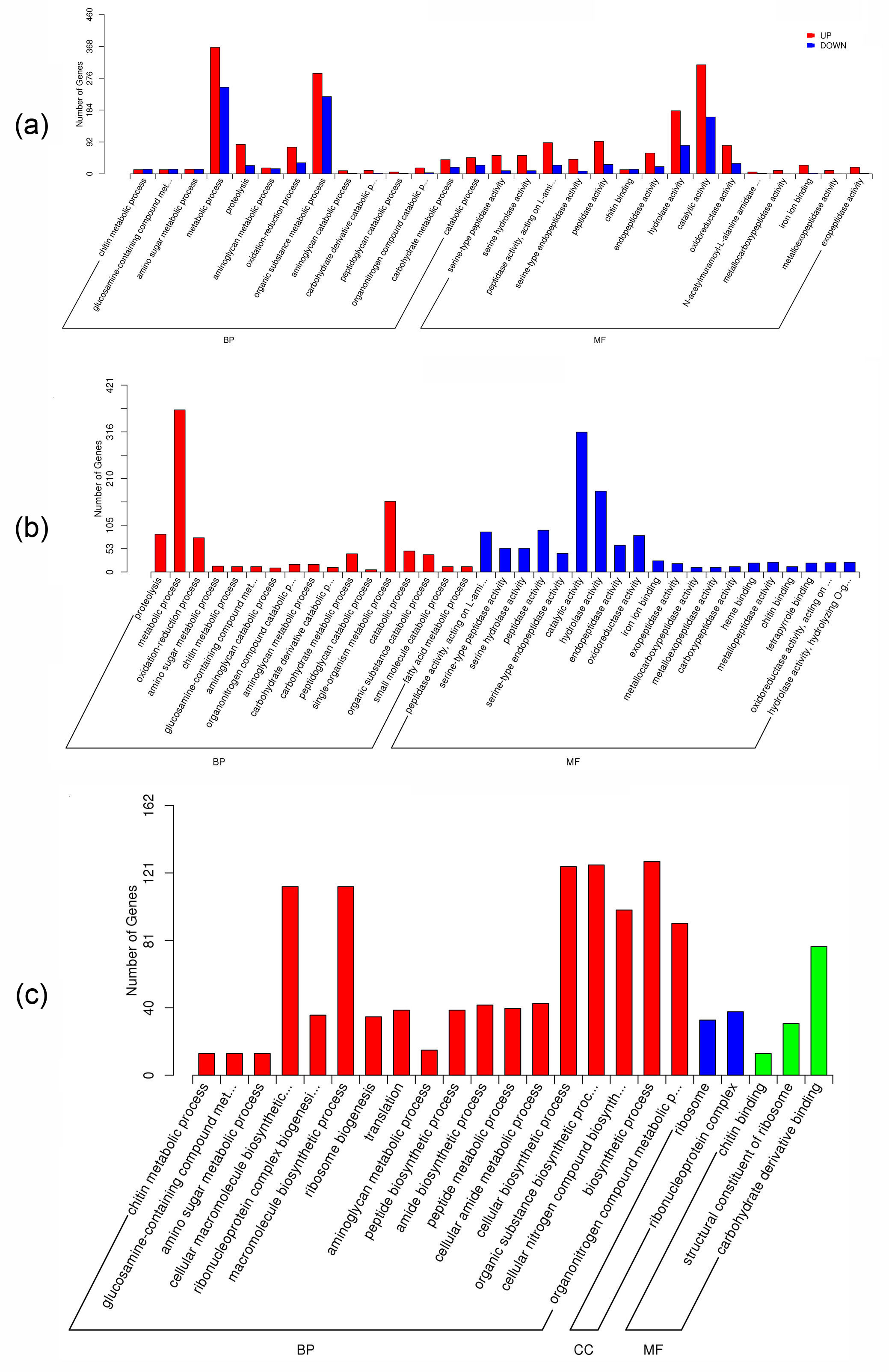


**Figure S5.** GO enrichment analysis DEGs (JCS vs RCS).(**a**) The up- and down-regulated DEGs. (**b**) The up-regulated DEGs. (**c**) The down-regulated DEGs. The x-axis shows the GO terms, and the y-axis indicates the number of genes.


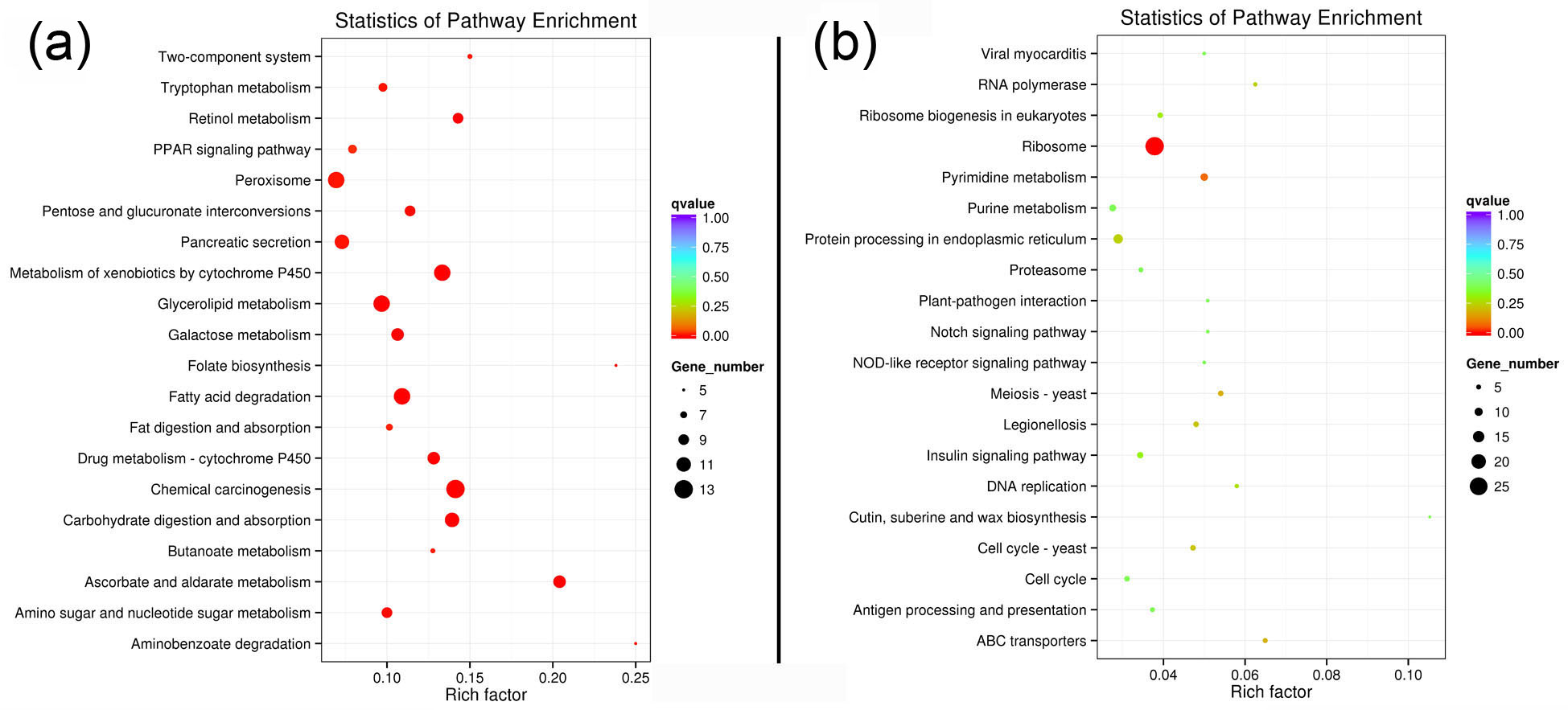


**Figure S6.** Scatterplot of enriched KEGG analysis of the up- and down-regulated DEGs. (**a**) The up-regulated DEGs. (**b**) The down-regulated DEGs. The enrichment factor indicates the ratio of the DEG number to the total gene number in a certain pathway. The color and size of dots indicate the range of *q*-value and gene number, respectively.
